# Walk-IT: An Open-Source Modular Low-Cost Smart Rollator

**DOI:** 10.3390/s22062086

**Published:** 2022-03-08

**Authors:** Manuel Fernandez-Carmona, Joaquin Ballesteros, Marta Díaz-Boladeras, Xavier Parra-Llanas, Cristina Urdiales, Jesús Manuel Gómez-de-Gabriel

**Affiliations:** 1 Ingeniería de Sistemas Integrados Group, Electronics Technology Department, University of Málaga—UMA, Complejo Tecnológico, 29071 Málaga, Spain; acurdiales@uma.es; 2 Department of Computer Science and Programming Languages, ITIS Software, University of Málaga—UMA, Complejo Tecnológico, 29071 Málaga, Spain; jballesteros@uma.es; 3 Technical Research Centre for Dependency Care and Autonomous Living—CETpD, Technical University of Catalonia—UPC, 08800 Barcelona, Spain; marta.diaz@upc.edu (M.D.-B.); xavier.parra@upc.edu (X.P.-L.); 4 Systems Engineering and Automation Department, University of Málaga—UMA, 29071 Málaga, Spain; jesus.gomez@uma.es

**Keywords:** rehabilitation robotics, assistive technology, smart rollator, gait analysis

## Abstract

Rollators are widely used in clinical rehabilitation for gait assessment, but gait analysis usually requires a great deal of expertise and focus from medical staff. Smart rollators can capture gait parameters autonomously while avoiding complex setups. However, commercial smart rollators, as closed systems, can not be modified; plus, they are often expensive and not widely available. This work presents a low cost open-source modular rollator for monitorization of gait parameters and support. The whole system is based on commercial components and its software architecture runs over ROS2 to allow further customization and expansion. This paper describes the overall software and hardware architecture and, as an example of extended capabilities, modules for monitoring dynamic partial weight bearing and for estimation of spatiotemporal gait parameters of clinical interest. All presented tests are coherent from a clinical point of view and consistent with input data.

## 1. Introduction

Nowadays, a significant percentage of the population present some form of disability. Disability may have a major impact in autonomy, especially when it affects mobility. Motor rehabilitation is of key importance to recover walking capabilities. Depending on their condition, people often rely on mobility aids such as rollators or canes to complete their rehabilitation therapy. During therapy, patient evolution can be assessed via gait analysis. Traditionally, gait analysis is visually performed by clinicians, based on their expertise. This process has been formalized into scale-based clinical assessment, i.e., clinical experts visually evaluate patients while they perform a number of tasks and fill out a clinical scale, such as the Tinetti mobility test [1]. Most scales are based on the way people walk and on how they support their weight on assistive devices, if any. Clinical scales usually return a global performance score rather than task-oriented scores and/or specific parameters such as stride length or step time. Scale-based assessment takes significant time from users and clinicians, so often it is only conducted at specific time instants, e.g., at the beginning and end of a rehabilitation process.

Technology offers alternatives to manually compiled clinical scales. A typical approach consists of using force/pressure sensors on a walking surface such as a treadmill or a walkway [2,3]. Optical motion capture systems [4,5,6,7,8] can provide more information, as specific body parts can be monitored during gait cycles. The main drawback of these approaches is that tests are constrained to specific locations, where specific equipment (and often expensive) is available and calibrated. In order to solve this issue, gait analysis has also been performed using wearable sensors, e.g., pressure sensors on feet soles [9], inertial sensors attached to the body [9,10], or even more intrusive ones, such as electromyographic electrodes attached to muscles of interest [11]. However, some of these solutions may not be comfortable for users, and sometimes sensors need to be calibrated and/or adapted to users by experts.

Alternatively, sensors can be attached to assistive devices rather than to specific environments or users themselves, so that monitorization is transparent to users and can be performed anywhere. Smart rollators are reportedly useful for monitorization purposes [12] as they are widely used in rehabilitation, and the structure may easily support all hardware and batteries. Smart rollators may automatically provide parameters of interest for gait analysis [13,14,15] and support estimation [16]. Some approaches rely on attaching Microsoft Kinect sensors [17] or Time of Flight (ToF) cameras [18] to a rollator frame to capture the user’s feet. However, Kinect sensors are sensitive to illumination and return a high bit stream and ToF cameras are expensive. The simplest approach to onboard sensor-based gait analysis is to attach force sensors to rollators handlebars [19,20], as users bear more weight on one or the other, depending on the foot they are using for support at the moment. This information is useful to detect heel strike and, then, derive other relevant gait parameters. The main drawback of this approach is that it is only reliable when users support significant weight on the rollator: Ballesteros et al. reported in [21] that peak differences between both handlebars during gait had to be larger than 7 N. While this condition is typically met by most users presenting mild to severe disabilities, the approach would not be valid for a significant number of target users, e.g., people with minor disabilities and/or at the end of their rehabilitation therapy.

A major problem with reported smart rollators is that most of them are ad hoc research prototypes, and commercial models are typically expensive. For example, the robotic rollator Guido had a large-scale commercial version with starting price over USD 6000 in 2004, which limited its success [22]. Besides, commercial systems are protected, so no modifications are allowed and any extra information required must be captured with independent wearable and/or external sensors.

This work proposes a new smart rollator, Walk-IT (designs publicly available as Supplementary software and hardware material at [23]), for gait monitorization to solve the commented issues. Its main contributions are that (i) it is an open-source modular system that can be deployed on any commercial nonrobotic rollator; (ii) modules can be added, replaced, and/or customized on a need basis, as all specifications and a Robot Operating System 2.0 (ROS2) control system are provided under a open-source license; (iii) it does not (necessarily) require any external sensors for monitorizarion, so it can be used anytime and anywhere.

The basic system already provides different gait-related parameters, e.g., step/stride time and length, or weight supported on the handles, but other parameters can be obtained by adding new modules. This work illustrates how partial weight bearing can be estimated while the user walks. This parameter is of interest because (i) it is important from a clinical point of view [24,25], and (ii) measuring support in clinical tests typically requires a fixed environments or direct expert intervention, and that may alter results [26].

This paper is organized as follows. First, Section 2 discusses clinical parameters of interest in gait assessment and how they can be obtained using the proposed smart rollator. Then, the overall system architecture is described in Section 3. Section 4 presents a dynamic partial weight-bearing approach based on the proposed structure and shows several preliminary experiments to show its capabilities, which are discussed in Section 5. Finally, conclusions and future work are presented in Section 6.

## 2. Gait Assessment Using a Smart Rollator

**Figure 1:**
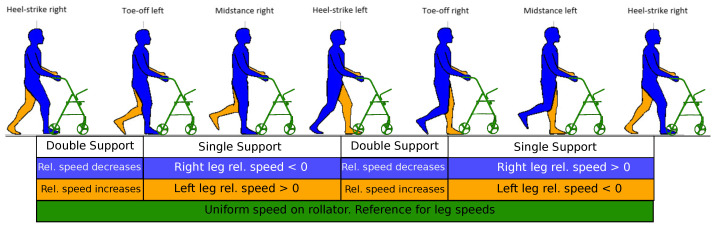
Leg support phases (single and double support) and velocities relative to moving rollator during gait cycle (Horizontal axis is not to scale).

The gait cycle (see Figure 1) describes periodic movements of the lower extremities that allow efficient body motion. This cycle can be divided into four phases depending on how much weight is distributed between both legs while walking. We have one single support phase per limb, plus two double support phases where load transitions from one leg to the other.

Single support phases usually comprise more than 60% of the cycle [27]. Clinicians rely on this particular phase to evaluate the partial weight-bearing during the rehabilitation process [24,25]. Rollators offer the most support in these phases, by decreasing loads on the support leg to reduce pain and improve balance. Additionally, they provide more accurate partial weight-bearing control to the patients [26].

There have been attempts at classification of gait abnormalities [28] based on how specific disabilities affect the gait cycle. Many works focus on disability impact on objective, measurable gait parameters. For example, users with antalgic gait are expected to improve weight-bearing through rehabilitation [29].

The authors already compiled most relevant studies to this respect in [21]. Specifically, the following parameters were found:

- Cadence (CAD): $\frac{steps}{min}$.
- Step time (SpT).
- Step length (SpL) *m*.
- Stride time (SdT).
- Stride length (Stance phase, SdL): *m*.
- Walking velocity (WV): $\frac{m}{s}$.
- Weight-bearing (WB): $N^{-1}$.

There are many works on average values for healthy individuals [30], and also on the impact of different disabilities on these parameters [31,32,33,34,35]. For example, patients with vestibular disabilities tend to increase CAD, decrease SdT, and decrease WV, i.e., they walk slower, with fast, short steps.

The authors also proposed in [21] a method to estimate spatiotemporal parameters on the i-Walker platform, a research prototype described in [36]. This platform included odometry and force sensors on the handlebars. In order to detect single support phases using only rollator onboard sensors, the process relied on calculating the force difference between the handlebars: when a person initiates heel contact, the handlebar force in the same side increases and the handlebar force in the opposite side decreases [20]. Steps were assumed to be the inflection points in the (filtered) force difference function ($f_{diff}$), and several parameters of interest could be extracted from the combination of this information and the rollator odometry:

- Step time (SpT): Average time between maximum–minimum (right) or minimum–maximum (left) in seconds.
- Stride time (SdT): Average time between maximum–maximum (right) and minimum–minimum (left) in seconds.
- Number of Step (NoS): Numbers of inflection points.
- Cadence (CAD): $60*\frac{NoS}{Tr}$.
- Step length (SpL): Average length between maximum–minimum (right) or minimum–maximum (left) in seconds.
- Stride length (SdL): Average length between maximum–maximum (right) and minimum–minimum (left) in seconds.
- Distance(d): Distance walked by user in meters.
- Average walking velocity (WV): $\frac{d}{Tr}$.

In addition, user’s support (UrS) was estimated as the sum of forces for all left or right steps and weight-bearing (WB) as the inverse of UrS.

The main drawback of this approach is that, as reported, it was only valid if users loaded significant weight on the rollator [37]. This problem could be partially solved by improving the platform sensory equipment. However, i-Walker was not an open system, so improving existing onboard sensors (odometry, for example, was not precise enough) was not possible. Furthermore, although onboard sensor readings could be extracted and combined with new sensors, integration and synchronization was a complex procedure.

This paper proposes a solution to both issues. First, as commented, we have developed an open smart rollator, where sensors can be easily replaced or added and the system operates under ROS, so integration of new modules is simple. Using this new open rollator, we can choose suitable odometry and force sensors regarding precision and also add new sensors for better single support phase detection.

The key idea under the new algorithm is that during the single support phase, one of the legs does not move, whereas the other is in a swing phase, as both legs cannot move simultaneously during the walk cycle. Hence, if both legs are identified and their speed is estimated, the still extremity will be the supporting one. As sensors are mounted on the rollator, taking its frame as reference, the still limb appears to move backwards (i.e., separate from the rollator), whereas the swinging limb moves forwards. Thus, the supporting limb can also be detected. We have added a 360${}^{{}^{\circ}}$ laser module to the basic Walk-IT configuration (odometry and force sensors) to calculate leg speed. Once the support limb is detected, partial weight-bearing can be calculated on the fly as the difference between the user’s weight and load supported on the rollator handlebars and all reported spatiotemporal parameters can be obtained as well.

As heel contact detection does not depend on weight-bearing on the platform, this new spatiotemporal detection algorithm solves the issues of the one in [37]; also, it will prove the flexibility of the proposed platform to add and/or replace new hardware and integrate it into its software architecture.

## 3. System Architecture

Walk-IT modules have two design guidelines. First, they should be compatible with standard commercial rollators and based on low-cost commercial components, so building one or several platforms becomes feasible anywhere. In addition, the original rollator frame must be changed the least and its support and mobility properties must not be affected, as these devices are certified for medical use and any significant modification would require a new certification process.

**Figure 2:**
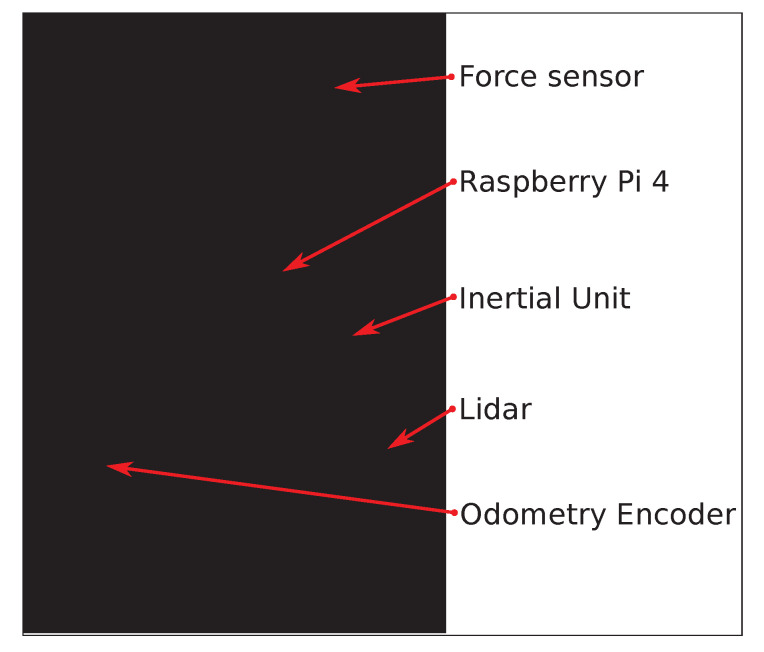
Kmina Comfort Rollator with proposed sensors and devices.

The Walk-IT in this work has been built over a Kmina Comfort Rollator [38] (Figure 2), a standard and affordable rehabilitation device. Sensors have been encased in plastic modules, attached to the frame, and connected to a Raspberry Pi 4 Model B, which runs the architecture. All electronics are powered by a USB power-bank, which can be charged as a regular USB home appliance. The system as a whole has been designed to look like an standard rollator.

Commercial sensors (e.g., laser) can be replaced by similar ones, and components of sensors specifically developed for Walk-IT (odometry, handle force measurement) are also widely available and easy to purchase from different manufacturers. As a whole, depending on the cost of desired attached sensors, a basic Walk-IT can be built for less than EUR 350 (assuming the original rollator frame to be around EUR 100). The following subsection describe each module in detail, as well as the ROS2 modules developed for basic control.

### 3.1. Hardware Modules

**Figure 3:**
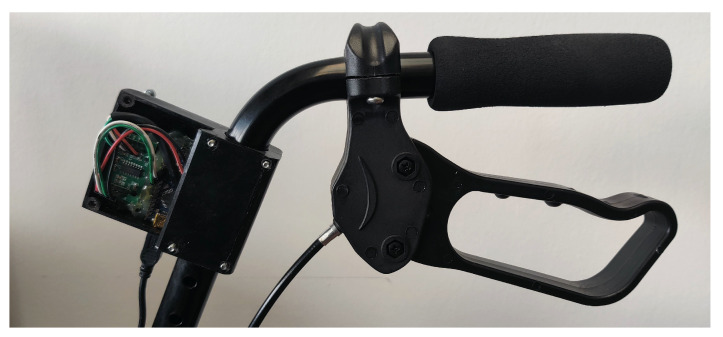
Handle sensor detail.

**Figure 4:**
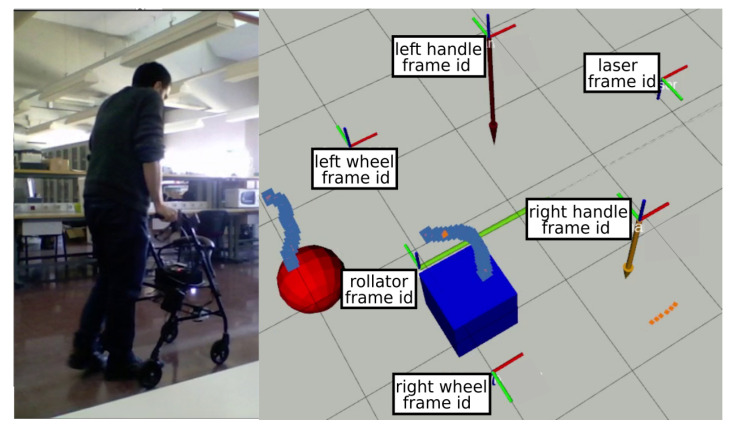
Live data-capture screenshot. Handle forces are displayed as vectors proportional to the load. Odometry is also shown as a green arrow at the middle point between rear wheels.

**Figure 5:**
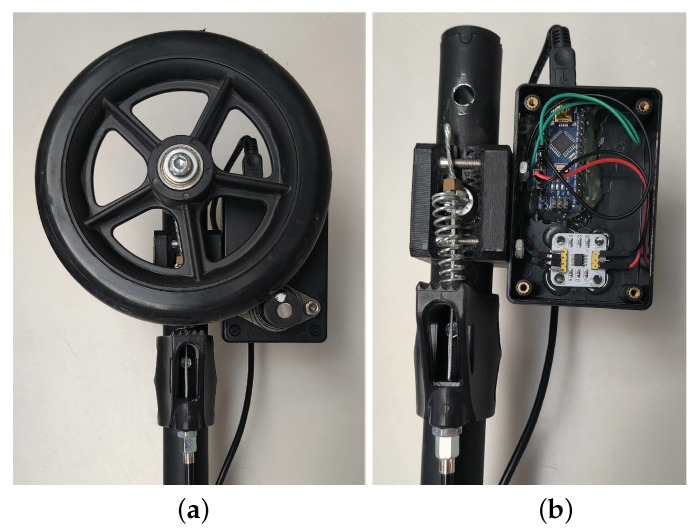
Encoder sensor: (**a**) encoder-wheel detail, (**b**) arduino encoder board.

Basic gait parameters includes cadence, walking velocity, or stride time and length, which may change depending on the type of maneuver. Walk-IT can directly measure rollator position, speed, and applied forces. Other parameters need to be derived from these ones via processing. In order to obtain these basic parameters, every Walk-IT rollator must include the following modules (others can be added on a need basis):

- Load sensors on the handlebars. Specifically, strain gauges are used in a Wheatstone bridge configuration to measure exerted support on each handle during the gait cycle. Gauges are adhered to the bars (Figure 3) and connected to an Arduino Nano using an HX711 24 bits Analog-to-Digital converter, specific for industrial control applications to interface directly with a bridge sensor such as the gauges. Figure 4 shows how load data is displayed under ROS2 with vertical arrows proportional to the applied force on their respective sensor.
  The Arduino board processes raw readings and makes them available through a serial-USB connection with a 2.5 Hz rate and a resolution of 5.5 gr. The whole circuit is protected with an adapted 3D-printed box attached to the bars. No modification of bar properties is performed, weight modification in the structure is minimal and symmetrical, and wiring is added to the brakes’ wiring harness. A basic Walk-IT includes one of these modules on each handlebar, to measure weight supported on each side of the rollator.
- Encoders on the wheels. User speed and step length are also required for gait analysis. Instead of modifying wheels in any way, encoders are attached to the rear wheels of the rollator, as shown in Figure 5. These encoders are built using an AS5601 magnetic rotary position sensor, protected with a plastic box and connected to an Arduino nano board via USB port. Encoders provide data at 600 Hz with an angular resolution of 0.1 mm per encoder tick. Again, no structure modification nor significant weight increase is required. The plastic box is positioned behind the frame to avoid interfering with patient’s movements while walking. Figure 4 presents odometry data as a green arrow displayed at the motion center. A basic Walk-IT includes one of these modules on each rear wheel, to provide right and left odometry.

Both proposed modules are connected via USB to a Raspberry Pi, where the control system is running under ROS2. Even though Raspberry Pi offers other hardware buses (I2C, UART...) suitable for communication with Arduino boards, USB is favored to allow potential upgrade/replacement from Raspberry Pi to different embedded systems if necessary.

Although the basic Walk-IT configuration already provides several gait parameters, to show how it can be modularly extended, an additional commercial sensor has been included in this work:

- Light detection and ranging sensor. A Light Detection and Ranging (LiDaR) has been attached under the lower-front transversal pole. This sensor has a twofold purpose: (i) to detect feet movement and gait phases, and (ii) to detect nearby obstacles and help with robot localization. In this work, an RPLidar A1M8 LiDaR from SLAMTEC was chosen. A1M8 is a low-cost LiDaR with 360 degrees field of view, 6-meter range, and an average scan rate of 5.5 Hz. Although it is required for leg support estimation, it is not strictly necessary for the basic configuration of Walk-IT.

It can be noted that, since Walk-IT runs on ROS2, new commercial sensors can be added directly to the architecture, while ad hoc sensors (handlebars, odometry) are simple to develop as well, given the variety of available libraries in ROS2.

### 3.2. Software Modules

Walk-IT architecture runs on the ROS2 framework [39], which includes a set of open-source software libraries and tools for robotic applications. ROS2 provides resources ranging from drivers for commercial sensors (e.g., LiDaR) to state-of-the-art algorithms (e.g., tracking and localization), as well as multiple developer tools for different programming languages and operating systems. Additionally, ROS2 also allows real-time performance constraints, aiming for industrial-grade applications [40]. ROS2 structure simplifies sharing and reusing software and extending modular systems.

Although the Walk-IT rollator in this work does not move autonomously, ROS2 has been selected because it provides a solid architecture style and communication mechanisms, as well as drivers for most commercial robot hardware. Furthermore, it simplifies evolution for future Walk-IT versions that also provide physical assistance.

**Figure 6:**
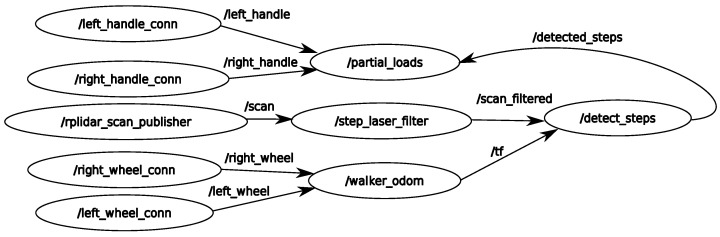
Main ROS2 nodes and topics.

Figure 6 summarizes the implemented Walk-IT ROS2 infrastructure. Software modules are represented as *nodes*. Nodes on the left are linked to onboard sensors, namely, right and left handle gauges, right and left odometry sensors, and the LIDAR. Nodes corresponding to commercial sensors are programmed using drivers provided by manufacturers, whereas new drivers for Walk-IT ad hoc hardware under ROS2 have been developed. These nodes publish data as *topics* to the system (represented by arcs), to which other nodes may subscribe.

**Figure 7:**
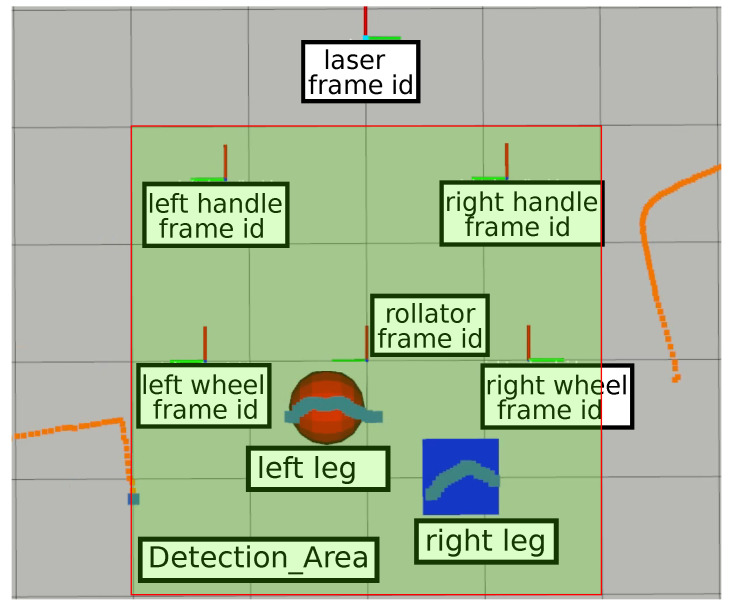
Live data capture screenshot. Legs are detected within the detection area and labeled using filtered laser data.

It is important to note that these nodes may use different coordinate systems, so the architecture relies on the *tf2* ROS2 library for tracking and management of multiple coordinate frames over time. The ROS2 *tf2* library keeps track of location data in a buffered tree structure, allowing transformation between any two coordinate frames at any given time. Figure 7 shows the six frame coordinates used in the presented Walk-IT implementation (x-axis in red and y-axis in green). The different coordinate systems are centered on the laser location, left and right handlebars, left and right wheels, and base footprint (foot between the rear wheels axis).

Using available captured data, the rest of the nodes process information to estimate where the user’s feet are and, then, how much weight they are supporting on the support leg. First, the *step_laser_filter* node removes all laser readings outside a detection area centered around the rear side of the walker. This node is meant to detect readings corresponding to user’s legs, discarding readings from nearby obstacles. This filtering process relies on the ROS library *laser_filters*, which is highly efficient, makes the output available to any other nodes, and can be expanded with other filters if needed. Figure 7 shows the detection area. Laser readings inside this area are plotted in gray, whereas the rest are plotted in orange. Filtered data is then fed to node *detect_steps*.

Node *walker_odom* calculates odometry from the left and right wheel encoder sensors. This node works under the assumption that the rollator can be considered a differential drive, as front wheels are actually caster wheels. Differential drive kinematics allow to obtain translational and rotational speeds from wheel speeds, and, from those, current walker position.

**Figure 8:**
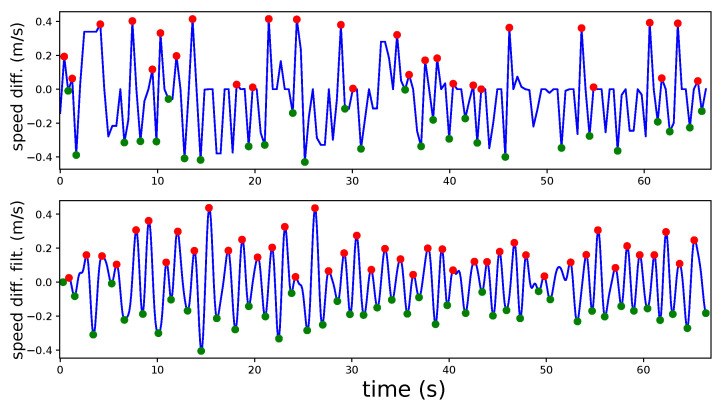
Contact heel detection (red/green dots) over leg $v_{diff}$ signal and over filtered version.

Node *detect_steps* uses the filtered laser scans to find legs’ position and speed. Figure 7 represents the left and right feet as a red circle and blue square, respectively. The LIDAR is fixed to the rollator structure, so all calculations are relative to the rollator frame coordinate system. The *detect_steps* node fits laser reading points within the detection area into two clusters—one for each leg—using OpenCV random forest classifier. After these clusters are established, leg positions are given by the centroids of the clusters. Legs are tracked over time to obtain their velocities $v_{left}$ and $v_{right}$. The velocity difference function $v_{diff}$ is filtered using a discrete Butterworth bandpass filter to remove noise and, finally, thresholding returns the peaks of the filtered function, which are heel contact instants for the left and right legs during gait. This process is illustrated in Figure 8, which shows detected peaks for both a random user unfiltered and filtered $v_{diff}$. Most peaks are detected but there are some false negatives (e.g., around seconds 15 and 30). These false negatives points may be due to steering points where the user’s legs are occluded by each other or by the rollator frame or other situations compromising leg detection. Signal filtering improves peak detection, as shown in the second plot of the figure. It can be observed that filtered $v_{diff}$ is cleaner and more peaks can be detected (e.g., less false negatives around seconds 15, 30, and 60).

Finally, weight on the support leg is the difference between user’s weight and supported weight detected on the handlebars. The node *partial_loads* is in charge of this task. Weight on handlebars is directly provided by topics *left_handle* and *right_handle*.

In order to implement the proposed spatiotemporal gait parameter algorithm, a new node is added to this basic architecture. This node simply subscribes to the output of the *partial_loads* node and uses system odometry to obtain the necessary values to calculate the spatiotemporal parameters as described in Section 2.

## 4. Tests and Results

This section presents preliminary results with eleven volunteers (Table 1) to illustrate the platform capabilities. Users were requested to make a round trip along a corridor without any other advice. The test group was balanced in gender, and age ranged between 30 and 90 years. Although balanced in age and gender, seven volunteers had no walking disabilities nor experience using rollators, whereas only one volunteer had wide knowledge of rollators and experience using them, from a past polytraumatism on the right side, and another three had some lesser conditions and knowledge of rollators.

### 4.1. Leg Speed Analysis

**Figure 9:**
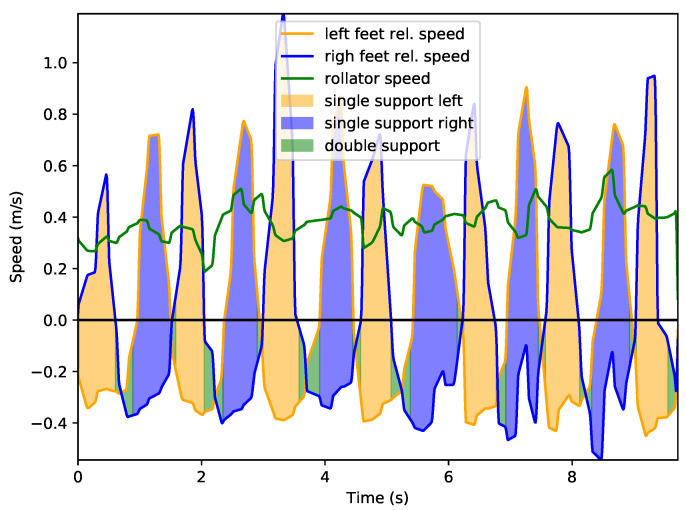
Leg motion speed relative to rollator for user 1.

**Figure 10:**
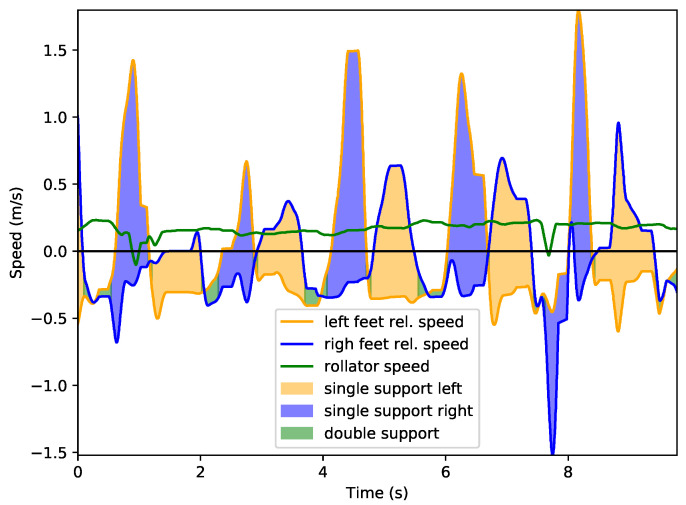
Leg motion speed relative to rollator for user 2.

In this section we will describe in detail how leg speed information can be used to assess gait parameters, focusing on a healthy user (1) and one with some existing condition (2). Figure 9 and Figure 10 present leg speed for users 1 and 2. Left and right feet are plotted in yellow and blue, respectively, and the rollator speed is presented in green. Speeds are calculated with respect to the rollator, so negative leg speeds mean that the support leg is left behind when the rollator moves forwards, as commented. This is even more noticeable during double support phases, when both legs show negative relative velocities. Areas below relative speed are also colored to provide information about leg support. Light yellow areas mark single support on left leg while light blue areas show support on right leg. Double support events are marked using light green. As described in Section 2, the supporting leg is always the *slowest* one in terms of the relative speeds.

It can be observed that user 1’s gait is quite symmetric, with minor, non-consistent changes from one leg to the other. Double support events are shorter with sharper transitions between feet. On the other hand, user 2 presents a lower, more homogeneous rollator speed, but significant gait asymmetries: his right leg presents a lower speed that the left and his right leg support time is significantly lower. Additionally, speed changes are smoother, with longer times spent in double support than with user 1. This is consistent with clinical evidence: users with this kind of disability tend to walk slower, more carefully, and support less weight on the affected extremity.

### 4.2. Partial Weight-Bearing

**Figure 11:**
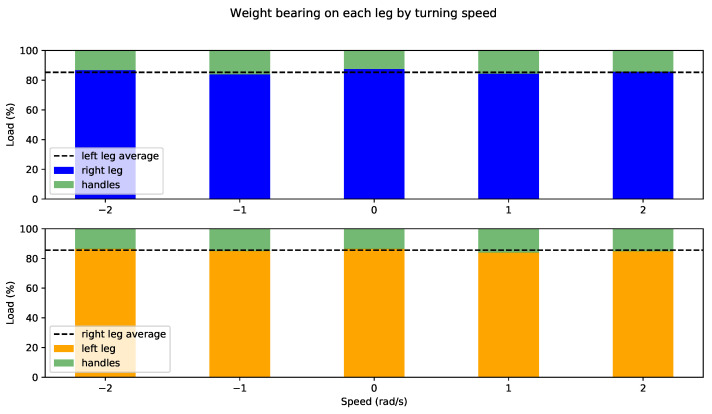
Weight bearing on support leg for user 1 by steering angle.

**Figure 12:**
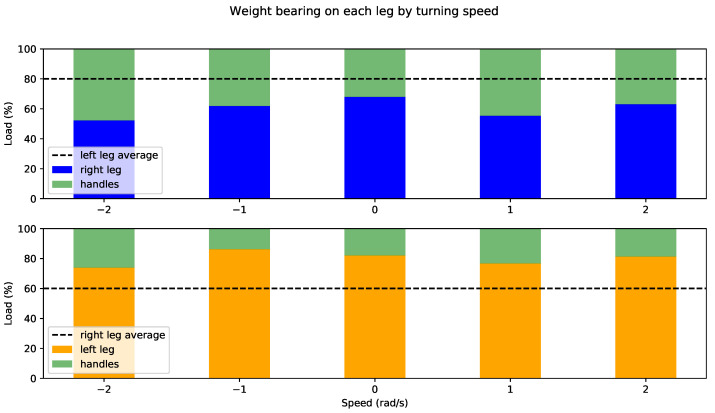
Weight bearing on support leg for user 2 by steering angle.

Once the support leg has been identified, the partial weight-bearing is obtained as the difference between the user’s weight and weight supported on the rollator handlebars depending on the maneuver, i.e., how fast they are steering the rollator. Figure 11 and Figure 12 show results for users 1 and 2, respectively. Support on left and right leg is represented in yellow and blue, respectively, whereas weight supported on (both) handlebars is represented in green in both plots. Each set of bars has a horizontal line corresponding to the average of the *opposite* leg load for convenience.

As expected from a user with no weakness in the lower limbs, user 1 supports very little weight on handlebars, plus variations from average are minimal for left and right leg and practically do not depend on the maneuver. User 2, on the other hand, presents major asymmetries. It can be observed in Figure 12 that average partial weight-bearing on the left leg is approximately 80%, which decreases to 60% on the right leg. Moreover, there are differences depending on the maneuver: user 2 supports less weight on sharper turns, relying more on the handles, especially steering left (negative turning speed). It can be also noted that this support increase is significantly more noticeable on the affected leg (right), as expected.

### 4.3. Spatiotemporal Gait Parameters Analysis

Finally, we present obtained spatiotemporal gait parameters for all volunteers in the test (Table 2). As most of our users were healthy or only presented a minor disability, on average parameters for left and right legs are mostly symmetrical and close to what should be expected from a healthy user (reported mean values [30] for healthy volunteers are CAD 110 steps/s, SdT 1.07 s, SdL 1 m, SpT and SpL 0.54 s and 0.72 m—symmetrical for both legs—and WV 1.33 m/s). In general, it was visually observed that volunteers moved slower than usual, probably because most of them had no experience at all with rollators. This observation is supported by average WV, which is only 0.72 m/s. For the same reason, it is important to note that volunteers 3, 5, 6, and 7 did not support much weight on the platform, so gait parameters estimation systems based solely on weight support would fail in these cases [37].

## 5. Discussion

Design of the proposed open license smart rollator has been optimized for (i) low cost; (ii) minor laterations to the original structure, and (iii) extraction of meaningful information from users’ gait using only onboard sensors. The first two goals have been achieved, because the whole system is under EUR 500 (rollator frame included) and, according to users and clinicians, there is no significant difference between using this smart rollator and a conventional one. In order to prove the third goal and also to test flexibility through modularity, partial weight bearing and spatiotemporal gait parameters have been extracted, as presented in the previous section.

Regarding partial weight bearing, persons with physical disability in their lower limbs reportedly support more weight on the rollator frane and have a symmetrical gait. This fact is supported by our tests in Section 4.2. Furthermore, the system provides information on which type of maneuvers require further weight-bearing (typically, sharp turns). Although this information was expected, it is important to note that the system provides quantitative measures of support for both legs on every maneuver and its evolution in time, so (i) the impact of rehabilitation therapies or degeneration processes can be assessed and (ii) punctual anomalies (outlayers) can be detected to act proactively (e.g., fall risk assessment).

Regarding spatiotemporal parameters calculation, as commented, the authors already proposed a method based uniquely on weight supported on handlebars, but it was only reliable when users supported significant weight on the rollator, i.e., persons with mild to severe physical disabilities. Using the Walk-IT rollator and the proposed laser-based method, spatiotemporal gait parameters are consistent for all users. In general, average SdL is a bit smaller than its reference value (0.63/0.72 m), but presents significant variance among users that may depend on their height (taller users have longer strides, but this value also depends on their condition). SdT, on average, is smaller than for healthy people (not using rollators), meaning that they are taking more steps to cover similar distances. Nevertheless, it must be noted that, again, there are significant variations among users. As they all are mostly healthy, not used to rollators, and were asked to bear weight in the device, results are not fully consistent, but, in general, younger people had a higher cadence (unless they had some minor condition) and faster strides. Additionally, although, as commented, gait was mostly symmetrical for both legs for all volunteers, differences were more noticeable for elderly adults.

Volunteer 2’s results deserve further analysis because, although he is in the younger group, he has the higher disability degree among all volunteers and large experience with rollators (although he does not need one anymore). His spatiotemporal parameters are consistent with a right leg injury, i.e., his stride length is low for his age and height, his steps are very regular (very low variation), and his right steps are slower than the left ones.

Finally, it needs to be noted that results for volunteer 6 are not coherent for her age, height, and condition, nor with visual observation of her test. This probably happened because she was the only volunteer wearing a long skirt that, most likely, interfered with the leg detection algorithm.

Future work will focus on two important aspects: (i) to establish a sound benchmark to validate key parameters of interest, and (ii) clinical validation of the current Walk-IT implementation with volunteers presenting different disabilities in hospitals and/or residences. We expect to achieve the first goal by using a reliable motion capture system in parallel with the rollator to establish a ground truth. Validation in hospitals requires safe, healthy conditions and approval of tests by their ethical committee, which largely depends at the moment on COVID-19 evolution. One limitation for the proposed gait analysis algorithm is that it relies on detecting the legs within the laser detection range. We will explore ways to fuse laser-based step detection with other sources to improve the overall reliability of the gait assessment.

We are also interested on extending Walk-IT capabilities to provide physical assistance to users by adding new hardware modules to adaptively brake the wheels (passive assistance).

## 6. Conclusions

This work has presented the Walk-IT platform, a new, low-cost open-source smart rollator under ROS2. The main contributions of Walk-IT are as follows:

- A system designed for easy replicability. Walk-IT relies on open software and off-the-shelf commercial components that can be easily replaced by similar ones, and it can be mounted on any standard rollator frame. In addition, hardware modules have been designed to be added to the base structure, so its original properties are not affected.
- A fully modular system. Walk-IT modules can be deployed/replaced on a need basis depending on the target application. In the present work, the Walk-IT basic configuration has been adapted for partial weight-bearing assessment, i.e., to measure how much weight a given user loads on each leg. Dynamic weight distribution is a very important parameter in clinical rehabilitation.
- An spatiotemporal analysis tool. Spatiotemporal gait parameters are reportedly linked to condition, so a spatiotemporal gait parameter capture algorithm based on leg detection has been implemented by including an additional node in the proposed ROS2 architecture.

This work has also presented a preliminary validation process. The system was tested by 11 volunteers, and results were consistent with expectations reported in the state of the art. Although extensive clinical tests are required for validation, these results are consistent with clinical reports and show that (i) the Walk-IT architecture gathers information as expected, and (ii) it can be easily modified with new sensors and modules for further uses.

The main drawback of the proposed system is that laser-based gait analysis requires visibility of the user’s legs, so it is not valid for people wearing long skirts or dresses. This drawback is intrinsic to the algorithm, so the method needs to be improved by adding other sensors or by combining information with weight bearing for users who support enough weight on the handlebars.

## Figures and Tables

**Table 1:** Users in tests.

	Age	Weight	Height	Preexisting Conditions	Gender	Experience Using Rollators
User 1	42	94	1.74	None	Male	No
User 2	38	110	1.84	Right leg polytraumatism	Male	Yes
User 3	39	112	1.75	None	Female	No
User 4	70	79	1.80	None	Male	No
User 5	69	58	1.65	None	Female	No
User 6	75	56	1.63	Rheumatoid arthritis	Female	No
User 7	90	51	1.55	Osteoarthritis	Female	Yes
User 8	39	74	1.80	None	Male	No
User 9	31	60	1.80	None	Male	No
User 10	42	83	1.75	None	Male	No
User 11	35	54	1.69	Arthritis	Female	Yes

**Table 2:** User gait parameters.

User	CAD	SdT	SdL	rSpT	lSpT	rSpL	lSpL	WV	UrS
1	58.41	1.05 (0.14)	0.55 (0.03)	0.75 (0.18)	0.83 (0.26)	0.28 (0.01)	0.27 (0.02)	0.53	22.68
2	99.01	0.61 (0.01)	0.35 (0.01)	0.48 (0.02)	0.43 (0.01)	0.18 (0.00)	0.17 (0.00)	0.57	35.17
3	106.62	1.17 (0.19)	1.01 (0.21)	0.58 (0.19)	0.59 (0.07)	0.51 (0.21)	0.51 (0.11)	0.86	<1
4	80.91	0.75 (0.20)	0.47 (0.09)	0.53 (0.17)	0.61 (0.31)	0.19 (0.02)	0.27 (0.05)	0.62	24.49
5	89.36	0.67 (0.13)	0.38 (0.05)	0.55 (0.19)	0.46 (0.18)	0.22 (0.02)	0.16 (0.01)	0.56	8.37
6	95.17	0.64 (0.19)	0.83 (0.25)	0.52 (0.42)	0.44 (0.13)	0.46 (0.14)	0.37 (0.08)	0.72	5.83
7	89.67	0.68 (0.26)	0.45 (0.08)	0.53 (0.31)	0.48 (0.27)	0.27 (0.04)	0.19 (0.03)	0.66	6.56
8	119.42	0.51 (0.03)	0.85 (0.19)	0.39 (0.08)	0.38 (0.03)	0.44 (0.11)	0.4 (0.08)	0.96	11.56
9	106.15	0.57 (0.02)	0.71 (0.04)	0.44 (0.04)	0.42 (0.02)	0.39 (0.02)	0.32 (0.02)	0.91	17.05
10	96.91	0.62 (0.03)	0.78 (0.05)	0.46 (0.04)	0.46 (0.05)	0.40 (0.03)	0.37 (0.02)	0.75	23.01
11	85.94	0.71 (0.20)	0.56 (0.07)	0.47 (0.11)	0.61 (0.35)	0.23 (0.02)	0.34 (0.04)	0.79	12.66
Av.	93.41	0.72	0.63	0.51	0.52	0.32	0.31	0.72	15.21

## Data Availability

Not applicable.

## Supplementary Materials

Hardware designs and software developed in this work can be accessed and downloaded at https://github.com/TaISLab/WalKit/tree/foxy.
